# Trends in Hospitalization and Mortality for Influenza and Other Respiratory Viruses during the COVID-19 Pandemic in the United States

**DOI:** 10.3390/vaccines11020412

**Published:** 2023-02-10

**Authors:** Adeel Nasrullah, Karthik Gangu, Ishan Garg, Anam Javed, Hina Shuja, Prabal Chourasia, Rahul Shekhar, Abu Baker Sheikh

**Affiliations:** 1 Division of Pulmonology and Critical Care, Allegheny Health Network, Pittsburg, PA 15212, USA; 2 Department of Internal Medicine, University of Kansas Medical Center, Kansas City, KS 66160, USA; 3 Department of Internal Medicine, University of New Mexico Health Sciences Center, Albuquerque, NM 87106, USA; 4 Department of Internal Medicine, Allegheny Health Network, Pittsburgh, PA 15512, USA; 5 Department of Medicine, Karachi Medical and Dental College, Karachi 74700, Pakistan; 6 Department of Hospital Medicine, Mary Washington Hospital, Fredericksburg, VA 22401, USA

**Keywords:** respiratory syncytial virus, RSV, coronavirus 2019, COVID-19, influenza viruses, parainfluenza viruses (PIVs), human metapneumovirus (MPV), respiratory viral outbreak, national inpatient sample (NIS)

## Abstract

Seasonal epidemics of respiratory viruses, respiratory syncytial virus (RSV), influenza viruses, parainfluenza viruses (PIVs), and human metapneumovirus (MPV) are associated with a significant healthcare burden secondary to hundreds of thousands of hospitalizations every year in the United States (US) alone. Preventive measures implemented to reduce the spread of SARS-CoV-2 (COVID-19 infection), including facemasks, hand hygiene, stay-at-home orders, and closure of schools and local/national borders may have impacted the transmission of these respiratory viruses. In this study, we looked at the hospitalization and mortality trends for various respiratory viral infections from January 2017 to December 2020. We found a strong reduction in all viral respiratory infections, with the lowest admission rates and mortality in the last season (2020) compared to the corresponding months from the past three years (2017–2019). This study highlights the importance of public health interventions implemented during the COVID-19 pandemic, which had far-reaching public health benefits. Appropriate and timely use of these measures may help to reduce the severity of future seasonal respiratory viral outbreaks as well as their burden on already strained healthcare systems.

## 1. Introduction

The coronavirus disease 2019, caused by SARS-CoV-2 infection, was first reported in Wuhan, China, in December 2019 [1,2]. The United States (U.S.) reported its initial four cases on 28 February 2020, which exponentially increased in number in a very short span. The World Health Organization (WHO) declared COVID-19 infection a pandemic on 11 March 2020 [3]. SARS-CoV-2 is transmitted through exposure to respiratory fluids carrying the infectious virus via the following routes: (a) inhalation of very fine respiratory droplets and aerosol particles, (b) deposition of respiratory droplets and particles on exposed mucous membranes in the mouth, nose, or eye by direct splashes and sprays, or (c) touching mucous membranes with hands that have been soiled either directly by virus-containing respiratory fluids or indirectly by touching surfaces with the virus on them (fomites) [4]. Viruses affecting the respiratory tract, including respiratory syncytial virus (RSV), influenza viruses, parainfluenza viruses (PIVs), and human metapneumovirus (MPV) are also transmitted through similar mechanisms [5,6,7,8]. An immediate surge in preventive strategies such as hand hygiene, avoiding public gatherings, and wearing personal protective equipment was recommended by various preventive societies and government authorities [1,3]. These public health measures implemented to control the spread of the COVID-19 pandemic may have also changed the epidemiology of various other respiratory viruses sharing similar modes of transmission. Therefore, the purpose of this study is to examine the trends in hospitalization and mortality rates for various respiratory viral infections from January 2017 to December 2020 in the United States. This study aims to examine the impact of these infections on healthcare systems and communities by analyzing the number of hospitalizations and deaths caused by respiratory viral infections over a four-year period. By identifying trends in hospitalizations and mortality rates, this study will provide insight into the burden of these infections on the healthcare system and inform public health policies and interventions to mitigate the impact of these infections on communities. Additionally, this study will also provide important information on which respiratory viral infections are causing the most hospitalizations and deaths, which will be important in prioritizing efforts to prevent and control these infections.

## 2. Materials and Methods

### 2.1. Data Source

We utilized the National Inpatient Sample (NIS) from the Agency for Healthcare Research and Quality (AHRQ) from the years 2017 to 2020 [9,10]. The NIS is drawn from all states participating in the HCUP (Healthcare Cost and Utilization Project) and the sample accounts for more than 97% of the US population. The NIS approximates to a 20% stratified sample of discharges from US community hospitals, excluding rehabilitation and long-term care hospitals. Each NIS year includes over 7 million hospital stays.

### 2.2. Inclusion and Exclusion Criteria

All patients with an age ≥18 years from the United States who were admitted to the hospital with respiratory illness (influenza A/B, RSV, parainfluenza, or metapneumovirus) were included in the study. We used ICD-10 clinical modification (ICD-10-CM) codes to retrieve patient samples and comorbid conditions. For influenza A and B, the ICD-10 codes used were J10.XX, J11.XX, and J09.XX, and for COVID-19 infection U071, U00, U49, U50, U85, and J1282. For parainfluenza, RSV, and metapneumovirus, ICD-10 codes J12.2X, J12.1X, and J12.1X were used, respectively. Patients age <18 years with elective admission were excluded from the study. For the year 2020, co-infection with COVID-19 was excluded from the study.

### 2.3. Study Outcomes

The primary outcome was trends in hospitalization with respiratory illness from 2017 to 2020. Secondary outcomes were mortality trends in hospitalized patients.

### 2.4. Statistical Methods

STATA 17 (StataCorp L.L.C., College Station, TX, USA) was utilized for statistical analysis. As the NIS comprises a 20% stratified sample of discharges to extrapolate the results to the US population, weights provided by the NIS were utilized. The weighted sample was around 35 million discharges for each calendar year. Descriptive analysis was performed to determine the proportion for categorical variables using the survey command in STATA. Trends were calculated utilizing the trend weights provided with the data sample and rates were expressed per million admissions for that calendar year.

## 3. Results

From 1 January 2017 to 31 December 2020, the NIS data included 1,034,538 hospitalizations in the United States for influenza virus infection, 47,930 hospitalizations with an RSV infection, 19,880 hospitalizations with a PIV infection, 36,820 hospitalizations with an MPV infection, and 1,659,040 hospitalizations with a COVID-19 infection diagnosis listed during an inpatient episode of care. Here, we look at the hospitalization and mortality trends of these viruses.

### 3.1. Influenza Virus

**Figure 1:**
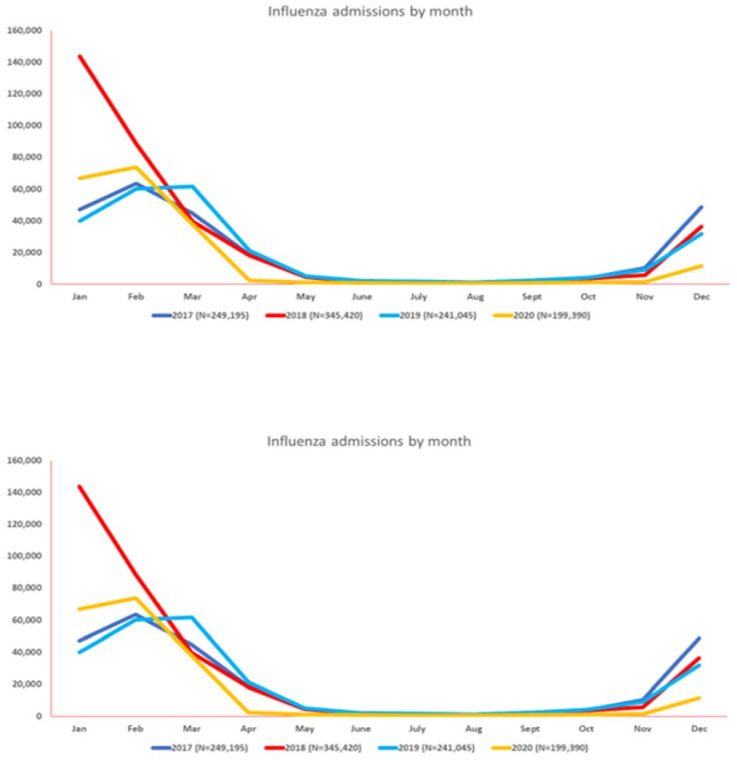
Monthly seasonal variation in the amount of influenza virus activity (hospitalizations at top, mortality at bottom) from 2017–2020.

The total number of hospital admissions for influenza infection by year was 249,069 (2017), 345,212 (2018), 240,949 (2019), and 199,308 (2020). Based on trends from January 2017 to December 2020, hospitalizations and mortality for influenza progressively increased from September onward until February to March, with the lowest admissions in August. The highest number of admissions and mortality for influenza were reported in 2018 as 345,420 and 13,145, respectively, and the lowest admissions and mortality in 2020 as 199,390 and 7310, respectively. Since the onset of the COVID-19 pandemic, the lowest rates of influenza admission were reported from April 2020 to December 2020 when compared to corresponding months from the prior three years. Figure 1 and Supplemental Tables S1 and S2 summarize the monthly hospitalization and mortality trends of the influenza virus from January 2017 to December 2020.

### 3.2. Respiratory Syncytial Virus (RSV)

**Figure 2:**
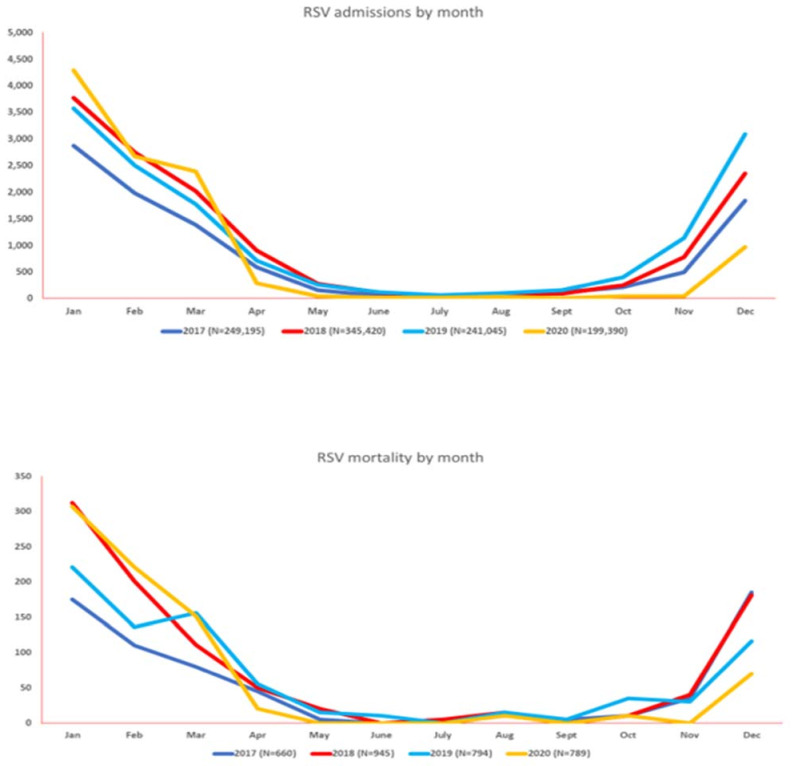
Monthly seasonal variations in the amount of respiratory syncytial virus (RSV) activity (hospitalizations at top, mortality at bottom) from 2017–2020.

The total number of hospital admissions for RSV by year was 9810 (2017), 13,420 (2018), 13,900 (2019), and 10,800 (2020). RSV hospitalizations and mortality progressively increased from September onward until February, with the lowest admissions in July. The highest number of admissions (13,900) was reported in 2019, and the highest mortality (795) was noted in 2019. The lowest admissions and mortality for RSV were noted in 2017 as 9810 and 660, respectively. Notably, before the onset of the COVID-19 pandemic, RSV had the highest rate of admission (38.58%) as compared to the past three years. However, after the onset of the COVID-19 pandemic, RSV had the lowest admission rates and mortality as compared to the corresponding months from the prior three years. Figure 2 and Supplemental Tables S3 and S4 summarize the monthly hospitalization and mortality trends of RSV from January 2017 to December 2020.

### 3.3. Parainfluenza Virus (PIV)

**Figure 3:**
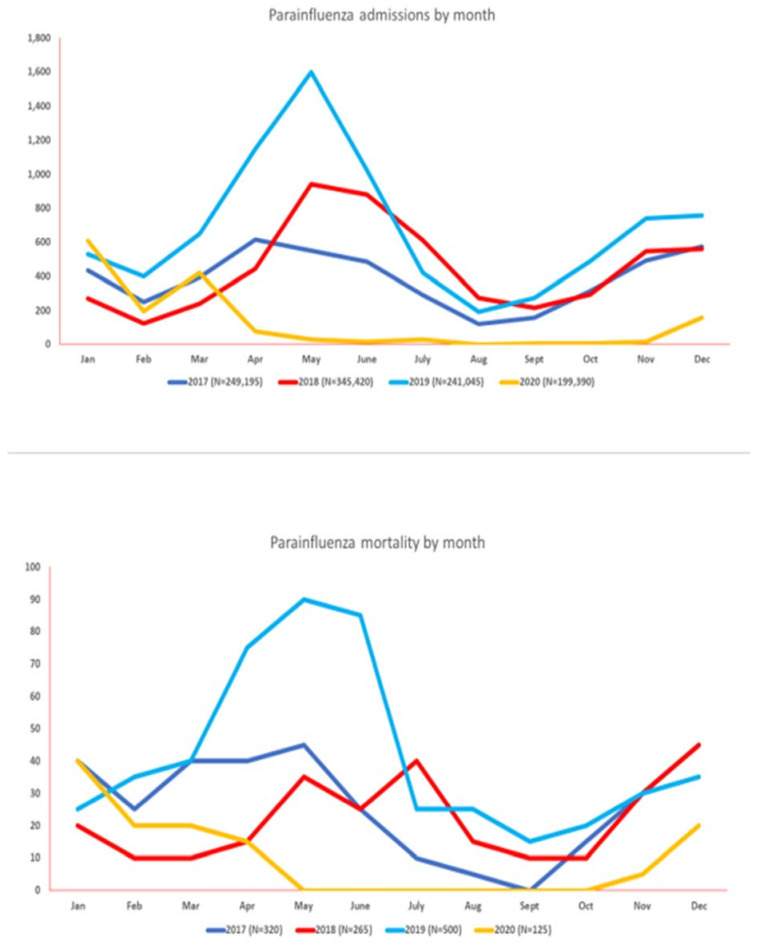
Monthly seasonal variations in the amount of parainfluenza virus activity (hospitalizations at top, mortality at bottom) from 2017–2020.

The total number of hospital admissions for PIV by year was 4685 (2017), 5400 (2018), 8235 (2019), and 1560 (2020). PIV was noted to have a variable pattern of infection but predominantly noted to have peak infections from April to June from 2017 to 2019. However, a significant rise in hospitalizations up to 38.90% was noted in Jan 2020, with the highest associated mortality (32%) from January 2017 to December 2020. After the onset of the COVID-19 pandemic, parainfluenza had the lowest admission rates and mortality as compared to the corresponding months from the prior three years. Figure 3 and Supplemental Tables S5 and S6 summarize the monthly hospitalization and mortality trends of PIV from January 2017 to December 2020.

### 3.4. Human Metapneumovirus (MPV)

**Figure 4:**
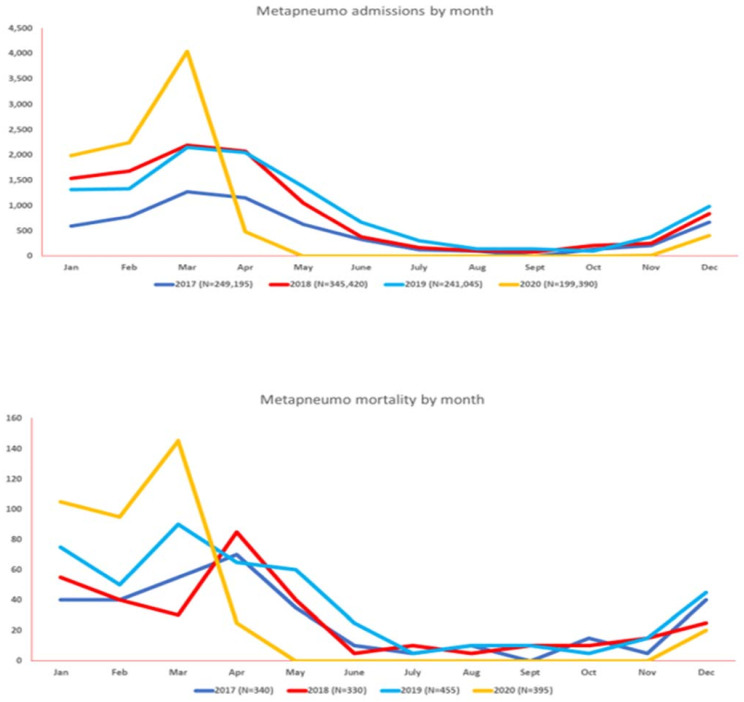
Monthly seasonal variations in the amount of human metapneumovirus (MPV) activity (hospitalizations at top, mortality at bottom) from 2017–2020.

The total number of hospital admissions for MPV by year was 6075 (2017), 10,570 (2018), 10,925 (2019), and 9250 (2020). MPV was noted to have peak infections predominantly from November to April (2017 to 2019). However, a significant rise in hospitalizations up to 43.66% was noted in March 2020, with the highest associated mortality (36%) from January 2017 to December 2020. After the onset of the COVID-19 pandemic, human metapneumovirus had the lowest admission rates and mortality as compared to the corresponding months from the prior three years. After the onset of the pandemic in March 2020, COVID-19 had an initial surge, with admissions up to 11%, which decreased to 5.65%. However, readmissions and corresponding mortality peaked again in November–December 2020. Figure 4 and Supplemental Tables S7 and S8 summarize the monthly hospitalization and mortality trends of MPV from January 2017 to December 2020.

### 3.5. COVID-19 Infection

**Figure 5:**
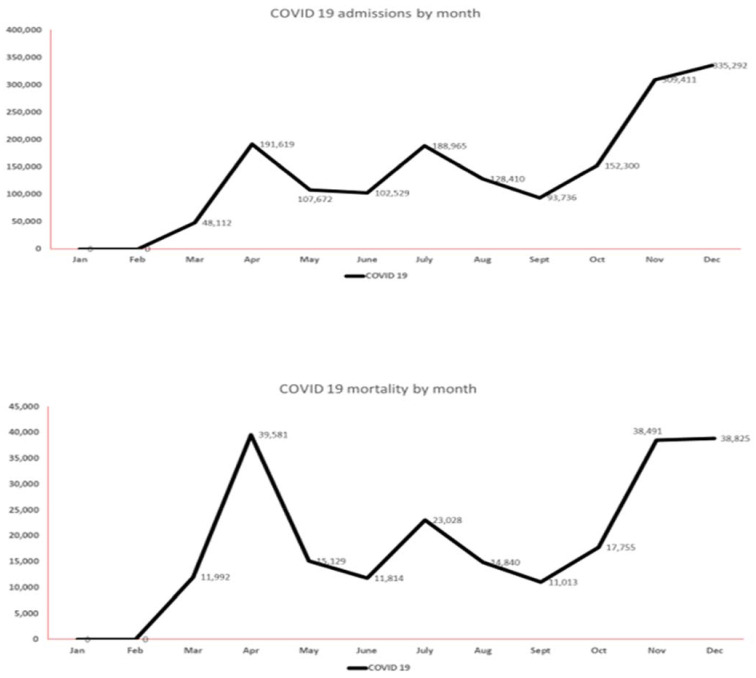
Monthly variations in the amount of COVID-19 activity (hospitalizations at top, mortality at bottom) during 2020.

The total number of hospital admissions for COVID-19 infection was 1,659,040 (2020). COVID-19 hospitalizations and mortality progressively increased from October to December (end of data analysis period), with the lowest number of admissions in the month of September. Figure 5 and Supplemental Tables S9 and S10 summarize the monthly hospitalization and mortality trends of COVID-19 from March 2020 to December 2020.

## 4. Discussion

To our knowledge, we report the largest amount of data from the US to date reporting the trends of various respiratory viral infections before the onset of the coronavirus disease-2019 (COVID-19) pandemic and until the rollout of the COVID-19 vaccine in December 2020. Findings from this study show that the onset of the COVID-19 pandemic (and the public health measures implemented as a result) had a significant impact on the transmission and epidemiology of other respiratory viruses, including the influenza virus, RSV, PIV, and MPV, with the lowest admission rates and mortality as compared to the corresponding months from the prior three years (2017–2019).

### 4.1. Seasonality and Future Outbreaks

Various respiratory viruses typically cause seasonal outbreaks between late fall and early spring. In the northern hemisphere, including the United States, this means an outbreak in the months of October–November to April–May, peaking around January–February [5]. The initiation of COVID-19 public health measures in 2020, including social distancing, personal protective gear (such as face masks), hand hygiene, and travel restrictions, coincided with the end of winter in the Northern hemisphere and the start of winter in the Southern hemisphere (start of respiratory viral infection season) [6]. In the US, influenza season spans from September to March every year, with an estimated 12,000 to 61,000 deaths annually since 2010 and a case fatality rate of 0.1% [7]. Influenza vaccine uptake was reported to be 35.4% in 2020–2021, which is the highest since 2016–2017 and relatively stable before the availability of COVID-19 vaccination in December 2020. Therefore, our study depicts the trends in influenza infections affected by non-pharmacologic interventions (NPIs) performed during the COVID-19 pandemic [8,9,10,11].

Our findings are consistent with the Influenza Hospitalization Surveillance Network from the US, which reported influenza-related hospitalizations as 0.8 per 100,000, the lowest since 2005 [12]. Alshami et al. reported their institutional data from New York, describing a decline in influenza positivity from 6.5% to 0.02% during the last six months of 2020, which is in parallel with our findings [13]. A nationwide Canadian study by Groves et al. reported data from a pediatric population that demonstrated a complete absence of influenza-related hospitalizations, intensive care unit admissions, or deaths [14]. Similarly, only one influenza-related pediatric death was reported in the US in 2020–2021, as compared to 144 in 2018–2019 [12]. A national database analysis from South Korea reported a robust termination of the influenza epidemic in 3 weeks with a 1.8–2.5-fold faster decline as compared to previous seasons [15]. Likewise, Suntronwong et al. from Thailand also reported significantly less influenza-like illness in 2020 as compared to 2019, with a similar decline in respiratory syncytial virus (RSV) [16]. Quandelacy et al. reported that until late September 2020, influenza A, RSV, and respiratory adenovirus incidence was lower as compared to 2012–2018 in southern Puerto Rico [17]. Synchronous results by Sullivan et al. from Australia reported a significant decline in the incidence of influenza [18]. In contrast, a decline in infectivity was reverted in places with poor compliance with NPIs. Sovann et al. reported an outbreak of influenza A in Cambodia in August 2020 because of the relaxation of national mitigation measures [19]. These significant downtrends in respiratory illnesses globally are likely attributed to public health interventions such as hand hygiene, universal masking, social distancing, restricting travel, screening, and isolation of sick individuals [12].

RSV infections were reported to have decreased by 20% since the onset of the COVID-19 pandemic [17]. In the United States, RSV monitoring is performed through the National Respiratory and Enteric Virus Surveillance System (NREVSS). As per the NREVSS, a notable decline in RSV-associated hospitalizations (0.3 per 100,000 persons) was noted in 2020 from October onward as compared to the prior season (27.1 per 100,000) [20], which is again in accordance with our findings. RSV is commonly associated with bronchiolitis. There was a significant reduction in hospitalizations in Brazil (>70% reduction) and Argentina during peak bronchiolitis season in 2020, which can likely be attributed to NPIs [21,22,23]. Similar findings showing decreased hospitalizations for RSV and flu were replicated from facility-based surveillance systems in South Africa [24]. Studies from New York did report a delayed peak in the RSV season in 2020, which again could be due to population-level immunity and delayed circulation [25]. Correspondingly, various countries have reported belated RSV outbreaks during the 2020–2021 season [4]. Since April 2021, a significant rise in cases of RSV has been noted, and it can likely be attributed to the recirculation of the virus due to minimal compliance with NPIs [5]. As of December 2022, the RSV hospitalization rate is 3.1 per 100,000 persons per month, which has risen significantly from prior years [20]. Prolonged absence of exposure to respiratory viruses may result in a lack of immunity, and as the circulation of viruses resumes, it may increase the severity of the disease in future RSV outbreaks [26,27]. This phenomenon is known as immunity debt. Supportive results have been reported by a number of studies. For instance, studies from Japan and Israel have a higher peak (with higher weekly case numbers and incidence rates) in the spring/summer of 2021 following a low incidence of RSV infections in 2020 [27,28].

Human metapneumovirus infections have remained low since the drop in admissions in April 2020. These findings are consistent with data reported by the CDC [5]. Similarly, parainfluenza infections remained low until the end of December 2020. According to the CDC, as of December 2022there is a significant rise in parainfluenza infectivity with all three serotypes, which explains the increased rate of parainfluenza-related hospitalizations [29]. Notably, we report crude mortality differences between COVID-19, influenza, and other respiratory viral illnesses. A French national database study by Piroth et al. described a higher severity of COVID-19 disease vs. seasonal influenza with an in-hospital mortality rate of 16.9% for COVID-19 vs. 5.8% for influenza [30]. Further discussion of mortality associated with each respiratory illness in comparison to COVID-19 is beyond the scope of this descriptive study.

Another important factor to consider in the altered balance of various viruses is virus interference, which refers to the phenomenon where one pathogen can affect the transmission or severity of another pathogen [31]. This can be mediated by various interferons with antiviral properties and RNA interference, especially developing in SARS-CoV2 [32]. Further research is needed to understand the long-term impacts of COVID-19 on the balance of other respiratory viruses. A combination of reduced human-to-human spread secondary to non-pharmaceutical interventions and changes in host and herd immunity may also have an impact on the evolution of these viruses. For example, lack of prior exposure and herd immunity may make future exposure more likely, which in combination with antigenic drift, may further limit vaccine efficacy and vaccine subtype mismatch [33]. These concerns are further compounded by a low level of circulating influenza virus strains, making it difficult to identify the ideal annual vaccine strain for the development of an effective vaccine.

### 4.2. Public Health Interventions

Our study shows the impact of non-pharmacologic interventions such as personal preventive strategies—including masking, social distancing, and personal hygiene—in decreasing the risk of hospital admission and crude mortality associated with influenza and other respiratory viral infections. Mandating masking in peak season through local and national authorities may mitigate transmission of viral respiratory illnesses and limit morbidity, mortality, and healthcare costs associated with these common respiratory viral illnesses. Moreover, vaccination against influenza for everyone aged six months or more should be emphasized [34]. It is also worth noting potential challenges in implementing these measures. For instance, infants and young children who are at higher risk for the development of severe symptoms from various respiratory pathogens may have more difficulty in being compliant with facemask protocols due to a combination of hyperactivity and poor-fitting masks. In addition, there are concerns that wearing a face mask may interfere with classroom learning and cognitive performance in children. Although large-scale studies on these concerns are lacking, a few smaller studies found no difference in cognitive performance between the mask-wearing vs. non-mask-wearing groups [35].

Vaccine hesitancy is a well-reported and major threat to the implementation of successful vaccination for the development of herd immunity. The WHO has categorized vaccine hesitancy as one of the top ten threats to global health, even prior to the COVID-19 pandemic. It is a complex issue requiring a multifaceted approach, including rebuilding public vaccine confidence through uniform, consistent, transparent, and effective communication and community engagement to improve vaccine acceptance [36].

The goal of any vaccination program is to achieve herd immunity. The herd immunity threshold depends on various factors such as basic reproduction number, vaccine, induced immunity duration, and viral transmission in a vaccinated individual. For example, assuming a basic reproductive number of four and vaccine efficacy of 80%, the community immunity level needs to reach at least 75% to stop the spread of an infection from becoming a pandemic [37].

Infection control (preventative) measures, including vaccines and non-pharmaceutical interventions such as masks, social distancing, and hand hygiene, may have an important impact beyond the viral pathogens studied in this review, including various potential pathogens causing respiratory tract infection that are spread through similar mechanisms, such as enterovirus D-68, adenoviruses, and rhinoviruses. According to some studies, unlike RSV, COVID-19-related preventative measures had only a limited impact on the spread of rhinovirus infection [4]. One of the postulated reasons is the longer survival of rhinovirus on external surfaces when compared to enveloped viruses such as COVID-19 and RSV [4]. It is also important to strategically employ these preventative measures to protect high-risk populations, including the elderly living in long-term facilities and infants in post-partum nursing care centers, infant care centers, and long-term care facilities.

### 4.3. Clinical and Diagnostic Tools

Viral respiratory panels have become the standard of practice across United States hospitals, allowing for quick and accurate diagnosis of common viral mono-or synchronous respiratory infections. With the ongoing COVID-19 pandemic and seasonal trends seen in the various respiratory viral pathogens, it is crucial to have diagnostic modalities to help differentiate between various viral agents presenting with overlapping clinical symptoms for optimized treatment and patient management. Some of the commonly available respiratory panel kits include Xpert Xpress SARS-CoV-2/flu/RSV (Xpert 4-in1) assay; Xpert Xpress SARS-CoV-2/flu/RSV; PowerChek SARS-CoV-2, influenza AandB, RSV Multiplex Real-time PCR Kit; and BioFire Respiratory Panel 2 [38,39,40,41]. 

### 4.4. Research and Surveillance Tools

Various modalities and measures were implemented during the COVID-19 pandemic—for example, large-scale surveillance data tools for a better understanding of disease spread—allowing for better allocation of public health resources and interventive measures. Development and utilization of similar tools for other viral respiratory tract infections may help provide timely and cost-effective public health interventions for preventing future loco-regional outbreaks. In addition, establishing a uniform global approach for viral genotyping for COVID-19 allowed for quicker collection and understanding of COVID-19 genomics and the development of safe and effective vaccines. A similar strategy can help in expanding genomic sequencing of respiratory viruses to allow a better understanding of viral diversity and evolutionary changes, which in turn can help in the development of effective and timely measures, including the development of effective vaccines.

### 4.5. Potential Public Health Implications

The practical implications of fewer viral respiratory illnesses due to masking, social distancing, and public health measures include a reduction in the number of hospitalizations and deaths caused by these illnesses. This can lead to a decrease in the burden on healthcare systems, including hospitals and healthcare workers, which can help to ensure that resources and personnel are available for other medical needs. Studies have shown reduced emergency department visits associated with respiratory illnesses and non-SARS-CoV2 viruses during the COVID-19 pandemic [12].

In addition, fewer respiratory viral illnesses can also lead to a reduction in absenteeism from work and school, which can have a positive impact on the economy. This can be especially important in times of economic uncertainty, such as during a recession. Furthermore, the implementation of social distancing, personal protective gear such as face masks, hand hygiene, and travel restrictions can also help to slow the spread of viral respiratory illnesses, which can prevent outbreaks and epidemics. This can be especially important for vulnerable populations such as older adults or people with underlying health conditions, who are at a higher risk for severe illness or death from respiratory viral infections. Moreover, these interventions can also have long-term health benefits, such as reducing the risk of chronic diseases associated with respiratory viral infections such as long-haul COVID, recurrent pneumonia, and bronchitis. Overall, the implementation of masking, social distancing, and public health measures can have a significant positive impact on public health and well-being by reducing the spread of viral illnesses and their morbidity and mortality.

## 5. Limitations

Our study has several limitations. The first is the inherent limitation due to the retrospective nature of the study. Secondly, the data was retrieved using ICD-10 codes and therefore is prone to coding errors. Moreover, uncontrollable factors such as barriers to health care during the COVID-19 pandemic were on the rise. Hence, it is plausible that some patients were infected with viral respiratory illnesses but did not seek care due to lockdowns or socioeconomic barriers. Additionally, testing for respiratory viral panels can be variable based on facilities and physician discretion. At present, NIS data is only available through the year 2020. The Omicron variant and further reported variants of COVID-19 were identified after 2020. It was therefore not possible to include those patients in this study. Although various measures and restrictions imposed during the COVID-19 pandemic had an impact on the circulation of various other viral pathogens, the exact extent of the impact of individual measures, such as face masks, social distancing, travel restrictions, etc., was not evaluated. Follow-up studies should be conducted to assess the impact of new variants of respiratory viral infections on mortality and hospitalization rates. Moreover, as this is database research, it lacks information about specific criteria for hospitalization for various respiratory viruses, and this should be acknowledged in interpreting the results. Regardless, we believe that the trends in hospitalizations and mortality rates for various respiratory viral infections in our study still provide useful insights into the burden of these infections on healthcare systems and communities. We hope that in the future additional studies can build upon these results and study the hospitalization and mortality trends of these respiratory viruses after the easing of COVID-19-related restrictions, among other questions.

## 6. Conclusions

Our study highlights that public health interventions for countering the spread of the COVID-19 pandemic significantly impacted the transmission of other respiratory viruses. We found a strong reduction in all viral respiratory infections, with the lowest admission rates and mortality in the last season (2020) compared to the corresponding months from the prior three years (2017–2019). However, since the advent and successful administration of the COVID-19 vaccine, there has been an easing of COVID-19-related restrictions. This, combined with immunity debt (i.e., lack of immunity in the community due to reduced exposure to infectious agents) may have increased susceptibility in the unexposed population to a more severe outbreak in future seasons. Therefore, implementation of public health policies mandating masking, vaccination, and other preventive measures, particularly during the peak season, may mitigate transmission of viral respiratory illnesses and limit morbidity, mortality, and healthcare costs associated with these common viral respiratory illnesses.

## Data Availability

Not applicable.

## Supplementary Materials

The following supporting information can be downloaded at: https://www.mdpi.com/article/10.3390/vaccines11020412/s1, Table S1: Influenza admissions by month from 2017–2020 with absolute number and percentages; Table S2: Influenza mortality by month from 2017–2020 with absolute number and percentages; Table S3: Respiratory Syncytial Virus (RSV) admissions by month from 2017–2020 with absolute number and percentages; Table S4: Respiratory Syncytial Virus (RSV) mortality by month from 2017–2020 with absolute number and percentages; Table S5: Parainfluenza admissions by month from 2017–2020 with absolute number and percentages; Table S6: Parainfluenza mortality by month from 2017–2020 with absolute number and percentages; Table S7: Human Metapneumovirus (MPV) admissions by month from 2017–2020 with absolute number and percentages; Table S8: Human Metapneumovirus (MPV) Mortality by month from 2017–2020 with absolute number and percentages; Table S9: COVID-19 admissions by month in 2020 with absolute number and percentages; Table S10: COVID-19 Mortality by month in 2020 with absolute number and percentages.
