# Supplementary material for: Trends in Hospitalization and Mortality for Influenza and Other Respiratory Viruses during the COVID-19 Pandemic in the United States
Source: Vaccines (Basel). 2023 Feb 10;11(2):412. doi: 10.3390/vaccines11020412 (PMC9966237; doi:10.3390/vaccines11020412)
Supplement: Supplementary file 1 [file vaccines-11-00412-s001.zip › vaccines-2179418-supplementary.pdf]

Supplemental File

Supplemental Table S1. Influenza admissions by month from 2017–2020 with absolute number and percentages.

Year (Number)	Jan	Feb	Mar	Apr	May	June	July	Aug	Sept	Oct	Nov	Dec
**2017 (N=249195)**	47272 (19%)	63644 (26%)	44830 (18%)	18316 (8%)	4809 (2%)	2068 (1%)	1420 (1%)	1246 (1%)	2367 (1%)	4062 (2%)	10342 (5%)	48693 (20%)
**2018 (N=345420)**	143729 (42%)	88704 (26%)	39654 (12%)	18100 (6%)	4421 (2%)	1451 (1%)	967 (1%)	898 (1%)	1969 (1%)	3489 (2%)	5665 (2%)	36165 (11%)
**2019 (N=241045)**	39989 (17%)	60285 (26%)	61828 (26%)	21116 (9%)	4990 (3%)	2073 (1%)	1663 (1%)	1350 (1%)	2314 (1%)	4315 (2%)	9160 (4%)	31866 (14%)
**2020 (N=199390)**	66915 (34%)	73834 (38%)	37764 (19%)	2512 (2%)	1097 (1%)	798 (1%)	558 (1%)	618 (1%)	817 (1%)	1296 (1%)	1495 (1%)	11604 (6%)

Supplemental Table S2. Influenza mortality by month from 2017–2020 with absolute number and percentages.

Year (numbers)	Jan	Feb	Mar	Apr	May	June	July	Aug	Sept	Oct	Nov	Dec
**2017 (N=7955)**	1485 (19%)	2085 (27%)	1450 (19%)	610 (8%)	155 (2%)	60 (1%)	40 (1%)	30 (1%)	30 (1%)	140 (2%)	380 (5%)	1490 (19%)
**2018 (N=13145)**	5676 (44%)	3184 (25%)	1537 (12%)	615 (5%)	150 (2%)	39 (1%)	50 (1%)	45 (1%)	60 (1%)	150 (2%)	205 (2%)	1431 (11%)
**2019 (N=7505)**	1400 (19%)	1825 (25%)	1865 (25%)	775 (11%)	140 (2%)	30 (1%)	30 (1%)	35 (1%)	90 (2%)	125 (2%)	310 (5%)	880 (12%)
**2020 (N=7310)**	2230 (31%)	2470 (34%)	1583 (22%)	190 (3%)	40 (1%)	50 (1%)	20 (1%)	35 (1%)	50 (1%)	70 (1%)	50 (1%)	521 (8%)

Supplemental Table S3. Respiratory Syncytial Virus (RSV) admissions by month from 2017–2020 with absolute number and percentages.

Year (numbers)	Jan	Feb	Mar	Apr	May	June	July	Aug	Sept	Oct	Nov	Dec
**2017 (N=9810)**	2868 (30%)	1986 (21%)	1384 (15%)	592 (7%)	155 (2%)	50 (1%)	45 (1%)	55 (1%)	120 (2%)	216 (3%)	496 (6%)	1840 (19%)
**2018 (N=13420)**	3766 (29%)	2747 (21%)	2020 (16%)	907 (7%)	275 (3%)	105 (1%)	39 (1%)	90 (1%)	90 (1%)	246 (2%)	777 (6%)	2351 (18%)
**2019 (N=13900)**	3574 (26%)	2505 (19%)	1776 (13%)	712 (6%)	260 (2%)	115 (1%)	64 (1%)	100 (1%)	160 (2%)	400 (3%)	1144 (9%)	3087 (23%)
**2020 (N=10800)**	4292 (40%)	2672 (25%)	2386 (23%)	285 (3%)	44 (1%)	14 (1%)	15 (1%)	29 (1%)	5 (1%)	44 (1%)	40 (1%)	968 (9%)

Supplemental Table S4. Respiratory Syncytial Virus (RSV) mortality by month from 2017–2020 with absolute number and percentages.

Year (numbers)	Jan	Feb	Mar	Apr	May	June	July	Aug	Sept	Oct	Nov	Dec
**2017 (N=660)**	175 (27%)	110 (17%)	80 (13%)	45 (7%)	5 (1%)	0 (0%)	0 (0%)	10 (2%)	5 (1%)	10 (2%)	35 (6%)	185 (29%)
**2018 (N=945)**	312 (33%)	201 (22%)	111 (12%)	50 (6%)	20 (3%)	0 (0%)	5 (1%)	15 (2%)	0 (0%)	10 (2%)	40 (5%)	181 (20%)
**2019 (N=795)**	221 (28%)	136 (18%)	156 (20%)	55 (7%)	15 (2%)	10 (2%)	0 (0%)	15 (2%)	5 (1%)	35 (5%)	30 (4%)	116 (15%)
**2020 (N=790)**	307 (39%)	221 (29%)	151 (20%)	20 (3%)	0 (0%)	0 (0%)	0 (0%)	10 (2%)	0 (0%)	10 (2%)	0 (0%)	70 (9%)

Supplemental Table S5. Parainfluenza admissions by month from 2017–2020 with absolute number and percentages.

Year (numbers)	Jan	Feb	Mar	Apr	May	June	July	Aug	Sept	Oct	Nov	Dec
**2017 (N=4685)**	435 (10%)	250 (6%)	395 (9%)	616 (14%)	550 (12%)	485 (11%)	290 (7%)	120 (3%)	160 (4%)	315 (7%)	491 (11%)	575 (13%)
**2018 (N=5400)**	270 (5%)	125 (3%)	240 (5%)	445 (9%)	940 (18%)	880 (17%)	610 (12%)	275 (6%)	215 (4%)	295 (6%)	545 (11%)	560 (11%)
**2019 (N=8235)**	530 (7%)	399 (5%)	650 (8%)	1150 (14%)	1599 (20%)	1024 (13%)	420 (6%)	194 (3%)	274 (4%)	490 (6%)	740 (9%)	759 (10%)
**2020 (N=1560)**	607 (39%)	196 (13%)	421 (28%)	75 (5%)	30 (2%)	15 (1%)	30 (2%)	0 (0%)	5 (1%)	5 (1%)	15 (1%)	160 (11%)

Supplemental Table S6. Parainfluenza mortality by month from 2017–2020 with absolute number and percentages.

Year (numbers)	Jan	Feb	Mar	Apr	May	June	July	Aug	Sept	Oct	Nov	Dec
**2017 (N=320)**	40 (13%)	25 (8%)	40 (13%)	40 (13%)	45 (15%)	25 (8%)	10 (4%)	5 (2%)	0 (0%)	15 (5%)	30 (10%)	45 (15%)
**2018 (N=265)**	20 (8%)	10 (4%)	10 (4%)	15 (6%)	35 (14%)	25 (10%)	40 (16%)	15 (6%)	10 (4%)	10 (4%)	30 (12%)	45 (17%)
**2019 (N=500)**	25 (5%)	35 (7%)	40 (8%)	75 (15%)	90 (18%)	85 (17%)	25 (5%)	25 (5%)	15 (3%)	20 (4%)	30 (6%)	35 (7%)
**2020 (N=125)**	40 (32%)	20 (16%)	20 (16%)	15 (12%)	0 (0%)	0 (0%)	0 (0%)	0 (0%)	0 (0%)	0 (0%)	5 (4%)	20 (16%)

Supplemental Table S7. Human Metapneumovirus (MPV) admissions by month from 2017–2020 with absolute number and percentages.

Year (numbers)	Jan	Feb	Mar	Apr	May	June	July	Aug	Sept	Oct	Nov	Dec
**2017 (N=6075)**	590 (10%)	781 (13%)	1271 (21%)	1155 (20%)	630 (11%)	330 (6%)	120 (2%)	100 (2%)	85 (2%)	135 (3%)	210 (4%)	665 (11%)
**2018 (N=10570)**	1540 (15%)	1685 (16%)	2190 (21%)	2071 (20%)	1055 (10%)	379 (4%)	165 (2%)	105 (1%)	79 (1%)	205 (2%)	249 (3%)	840 (8%)
**2019 (N=10925)**	1312 (13%)	1331 (13%)	2151 (20%)	2046 (19%)	1375 (13%)	665 (7%)	299 (3%)	144 (2%)	140 (2%)	99 (1%)	375 (4%)	980 (9%)
**2020 (N=9250)**	1992 (22%)	2242 (25%)	4039 (44%)	485 (6%)	34 (1%)	5 (1%)	5 (1%)	5 (1%)	5 (1%)	15 (1%)	15 (1%)	405 (5%)

Supplemental Table S8. Human Metapneumovirus (MPV) Mortality by month from 2017–2020 with absolute number and percentages.

Year (numbers)	Jan	Feb	Mar	Apr	May	June	July	Aug	Sept	Oct	Nov	Dec
**2017 (N=340)**	40 (12%)	40 (12%)	55 (17%)	70 (21%)	35 (11%)	10 (3%)	5 (2%)	10 (3%)	15 (5%)	15 (5%)	5 (2%)	40 (12%)
**2018 (N=330)**	55 (17%)	40 (13%)	30 (10%)	85 (26%)	40 (13%)	5 (2%)	10 (4%)	5 (2%)	10 (4%)	10 (4%)	15 (5%)	25 (8%)
**2019 (N=455)**	75 (17%)	50 (11%)	90 (20%)	65 (15%)	60 (14%)	25 (6%)	5 (2%)	10 (3%)	10 (3%)	5 (2%)	15 (4%)	45 (10%)
**2020 (N=395)**	105 (27%)	95 (25%)	145 (37%)	25 (7%)	5 (2%)	0 (0%)	0 (0%)	0 (0%)	0 (0%)	0 (0%)	0 (0%)	20 (6%)

Supplemental Table S9. COVID-19 admissions by month in 2020 with absolute number and percentages.

Year (numbers)	Jan	Feb	Mar	Apr	May	June	July	Aug	Sept	Oct	Nov	Dec
**2020 (N=1659040)**	0 (0%)	0 (0%)	48112 (3%)	191619 (12%)	107672 (7%)	102529 (7%)	188965 (12%)	128410 (8%)	93736 (6%)	152300 (10%)	309411 (19%)	335292 (21%)

Supplemental Table S10. COVID-19 Mortality by month in 2020 with absolute number and percentages

Year (numbers)	Jan	Feb	Mar	Apr	May	June	July	Aug	Sept	Oct	Nov	Dec
**2020 (N=222490)**	0 (0%)	0 (0%)	11992 (6%)	39581 (18%)	15129 (7%)	11814 (6%)	23028 (11%)	14840 (7%)	11013 (5%)	17755 (8%)	38491 (18%)	38825 (18%)
